# Differential Regulation of* Escherichia coli fim* Genes following Binding to Mannose Receptors

**DOI:** 10.1155/2018/2897581

**Published:** 2018-05-22

**Authors:** William R. Schwan, Michael T. Beck, Chia S. Hung, Scott J. Hultgren

**Affiliations:** ^1^University of Wisconsin-La Crosse, La Crosse, WI 54601, USA; ^2^Center for Women's Infectious Disease Research, Washington University, St. Louis, MO 63110, USA

## Abstract

Regulation of the uropathogenic* Escherichia coli *(UPEC)* fimB* and* fimE* genes was examined following type 1 pili binding to mannose-coated Sepharose beads. Within 25 min after mannose attachment,* fimE* expression dropped eightfold, whereas* fimB* transcription increased about two- to fourfold. Because both* fim* genes encode site-specific recombinases that affect the position of the* fimS* element containing the* fimA* promoter, the positioning of* fimS* was also examined. The* fimS* element changed to slightly more Phase-OFF in bacteria mixed with plain beads, whereas UPEC cells interacting with mannose-coated beads had significantly less Phase-OFF orientation of* fimS *under pH 7 conditions. On the other hand, Phase-OFF oriented* fimS* increased fourfold when UPEC cells were mixed with plain beads in a pH 5.5 environment. Positioning of* fimS *was also affected by* fimH *mutations, demonstrating that the FimH ligand binding to its receptor facilitates the changes. Moreover, enzyme immunoassays showed that UPEC cells had greater type 1 pili expression when mixed with mannose-coated beads versus plain beads. These results indicate that, after type 1 pilus binding to tethered mannose receptors, the physiology of the* E. coli* cells changes to maintain the expression of type 1 pili even when awash in an acidic environment.

## 1. Introduction

Urinary tract infections afflict 10.5 million women in the United States each year and uropathogenic* Escherichia coli* (UPEC) are primarily responsible for these infections in humans [1]. UPEC pathogenicity is the result of the action of several virulence factors, although type 1 pilus expression is thought to be the chief virulence factor produced by UPEC, and it is the first to be confirmed by Molecular Koch's postulates [2]. Critical roles that type 1 pili play in the onset and maintenance of a urinary tract infection include adherence to mannose receptors on uroepithelial cells lining the urinary tract and a role in invasion into bladder epithelial cells [3, 4]. Moreover, type 1 pili are one of the most frequently observed pilus structures on* E. coli* cells isolated from the urinary tracts of infected patients [5–9] and microarray analysis demonstrated that* fim* gene expression increases over time in UPEC cells colonizing the urinary tracts of mice [10].

Expression of type 1 pili is the result of phase variation, where there is a switching between nonpiliated cells (Phase-OFF) and piliated cells (Phase-ON) [11]. Two site-specific recombinases are primarily involved in determining whether the bacteria are Phase-OFF or Phase-ON by influencing the position of a* fimS* invertible element that contains the promoter for the structural gene,* fimA*. These recombinases include the FimB protein that allows switching from Phase-OFF to Phase-ON and FimE that promotes switching from Phase-ON to Phase-OFF [12–14]. Other site-specific recombinases have auxiliary roles in positioning of the* fimS* invertible element (reviewed in [15]). The growth environment can also have a substantial role to play in the ability of* E. coli* cells to phase vary and express type 1 pili. Modulation of type 1 pilus expression occurs as a result of changes in pH, temperature, the presence of aliphatic amino acids, glucose effects, and osmolarity [16–26].

No one has directly examined whether* fim* genes are regulated in some manner following the attachment of type 1 piliated UPEC cells to mannose receptors. Our previous work has indicated that capsule gene expression is adversely affected by UPEC cell FimH attachment to mannose-coated beads [27], and previous microarray work hints that* fim* gene expression could also be activated following ligand-receptor binding [10]. In this study, we have examined if* fimB* and* fimE* transcription are affected by the interaction between the FimH ligand and its mannose receptor. We demonstrate that* fimB* transcription is upregulated and* fimE* transcription is downregulated following the binding of FimH expressing UPEC bacteria to mannose-coated beads. Furthermore, the positioning of the* fimS* invertible element changes to a more Phase-ON orientation and more type 1 pili are produced following the FimH tip adhesin binding to mannose, suggesting that type 1 piliated UPEC cells change physiologically after attachment to the mannose receptors to maintain the adherence through a sustained commitment to type 1 pilus expression.

## 2. Materials and Methods

### 2.1. Bacterial Strains, Plasmids, and Growth Conditions

The NU149 uropathogenic strain of* E. coli* [28] was grown in Luria broth (LB) as previously described [6] to allow for optimal expression of type 1 fimbriae. The FimH mutants have been described previously [29]. Briefly, they represent site-directed mutants of the* fimH* gene of UPEC strain J96 cloned onto the pMMB66 plasmid. Plasmid pWS145-38 was also used and carries the* fimB* promoter region joined to a promoterless* lux* operon on a single copy number plasmid [25].

### 2.2. Binding to Plain or Mannose-Coated Sepharose Beads

The assays were performed as previously described [27]. Briefly, several tubes were set up, each with one aliquot of bacteria mixed with either Sepharose 4 L beads (Sigma Chemical Co., St. Louis, MO) or mannose-coated Sepharose beads [30]. After different time points, total RNAs were isolated from both populations using a hot phenol extraction procedure [31] and treated twice with RNase-free DNase (Boehringer-Mannheim) to remove contaminating DNA. Next, cDNAs were synthesized from 6 *μ*g of total RNA from each time point as previously described [32] by using the random hexamer primer from a reverse transcription- (RT-) PCR kit (Stratagene, La Jolla, Calif.). Alternatively, strain NU149/pWS145-38 cells were mixed with plain Sepharose beads, mannose-coated Sepharose beads, or mannose-coated Sepharose beads with 2% free D-mannose (wt/vol, Sigma) added in pH 5.5 or 7 LB with low osmolarity. Assays were performed at least three times on different days, and the data were expressed as means ± standard deviations.

### 2.3. Limiting Dilution-Reverse Transcribed-PCR (LD-RT-PCR)

Total RNAs were extracted from the NU149 cells after 10, 25, 60, and 120 min and converted into cDNAs as noted above. Using these cDNAs as templates, limiting dilution-reverse transcribed-polymerase chain reactions (LD-RT-PCRs) were performed with the FimB1/FimB2, FimE1/FimE2, and FtsZ1/FtsZ2 primer pairs as described previously [24]. Briefly, the cDNAs were twofold serially diluted and each dilution was PCR amplified. Integrated DNA Technologies (Coralville, IA) synthesized all of the primers used in this study. Amplification products were analyzed on 1.5% agarose gels, comparing the populations reacted with plain Sepharose versus mannose-coated Sepharose beads. Assays were performed at least three times on different days with different RNA preparations used to make the cDNAs.

### 2.4. In Vitro Bioluminescence Assays

Each culture grown overnight in pH 5.5 and pH 7 LB was incubated with plain Sepharose beads or mannose-coated Sepharose beads with or without 2% mannose (wt/vol) with rocking and then tested for bioluminescence using a FB 12 bioluminescence single tube luminometer (Zylux Corporation). The luminescence results were reported as relative luminescence units (RLU) as described previously [26]. Colony forming unit (CFU) for each culture was calculated by plating aliquots of 10-fold serially diluted bacteria in phosphate-buffered saline (PBS) onto LA containing 12.5 *μ*g/ml of chloramphenicol and counting the colonies. The RLU values were divided by the viable counts to achieve RLU/CFU for each culture.

### 2.5. PCR Analysis of the 314 bp* fimS* Invertible Element DNA

Chromosomal DNAs were extracted and processed as previously described [23]. The DNAs were standardized, and the preparations were used for PCR amplification as described by Schwan et al. [31] with the INV/FIMA primer pair for the* fimS* Phase-ON orientation, FIME/INV for the Phase-OFF* fimS* orientation, and EcFtsZ1/EcFtsZ2 for detecting* ftsZ* transcripts also being previously described [24–26]. Multiplex PCRs were performed using all of the primer pairs. Phase-ON and Phase-OFF* fimS* PCR product band intensities were standardized to the* ftsZ* amplification product using ImageQuant software. For confirmation of* fimS* orientation differences, LD-PCR was done with the INV/FIMA and FIME/INV primers as previously described [24]. The analyses were performed at least three times with different DNA preparations.

### 2.6. Enzyme Immunoassay (EIA) Analysis of Type 1 Pili Levels

An EIA was performed on strain NU149 cells grown in pH 5.5 or pH 7 LB mixed with plain Sepharose, mannose-coated Sepharose beads, or mannose-coated Sepharose with 2% free D-mannose (wt/vol) added. The assays were performed right immediately after mixing the bacteria with the beads (0 h) mixing and then again after a 24 h incubation with the beads at 37°C incubation as previously described [24]. EIAs were performed at least three times for each condition, and the values given below are means ± standard deviation.

### 2.7. Statistics

Student's *t*-test was used to calculate statistical variation. *P* values < 0.05 were considered significant.

## 3. Results

### 3.1. Transcription of* fimB* and* fimE* Changes after Type 1 Pili Binding to Mannose Receptors

**Figure 1:**
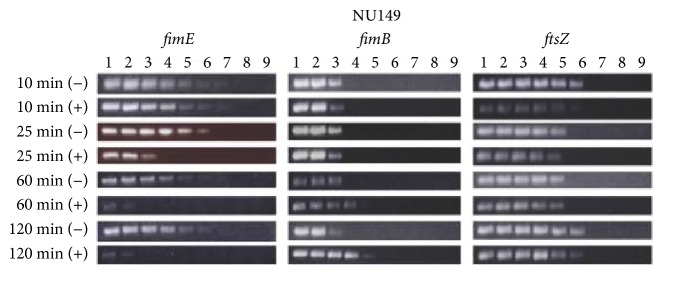
Quantitative determination of mRNA regulation by LD-RT-PCR analysis of cDNAs of strain NU149 cells mixed with plain Sepharose beads (−) or mannose-coated Sepharose beads (+) for 10, 25, 60, or 120 min. The FimB1/FimB2, FimE1/FimE2, and EcFtsZ1/EcFtsZ2 primer pairs were used to amplify serially twofold diluted cDNAs and targeted* fimB* (379 bp product),* fimE* (392 bp product), and* ftsZ* (302 bp product) transcripts, respectively. All PCR products were electrophoresed on 1.5% agarose gels. The following dilutions of cDNAs were used: undiluted (lane 1), 1/2 (lane 2), 1/4 (lane 3), 1/8 (lane 4), 1/16 (lane 5), 1/32 (lane 6), 1/64 (lane 7), 1/128 (lane 8), and 1/256 (lane 9). The data represent at least three separate runs.

To determine if type 1 pilus binding to mannose receptors affected* fim* gene expression, a LD-RT-PCR assay was performed. The results indicated that at the 10 min time there was no difference between the* E. coli* cell populations mixed with plain Sepharose compared to mannose-coated Sepharose. However, beginning at 25 min and proceeding through 120 min, there was a gradual decline in the level of* fimE* transcripts in the mannose-coated Sepharose population compared to the plain Sepharose population that culminated in a 16-fold decline after 120 min (Figure 1). The level of the control* ftsZ* transcripts remained unchanged throughout the time course for both plain and D-mannose populations. In addition, the level of* fimB* transcripts began to rise after 60 min and rose fourfold after 120 min compared to both the 10 min point and the 120 min time point that was mixed with plain Sepharose. This suggested that the ligand-receptor interaction between type 1 pili and the mannose receptors led to the downregulation of* fimE* transcription and an activation of* fimB* transcription.

**Figure 2:**
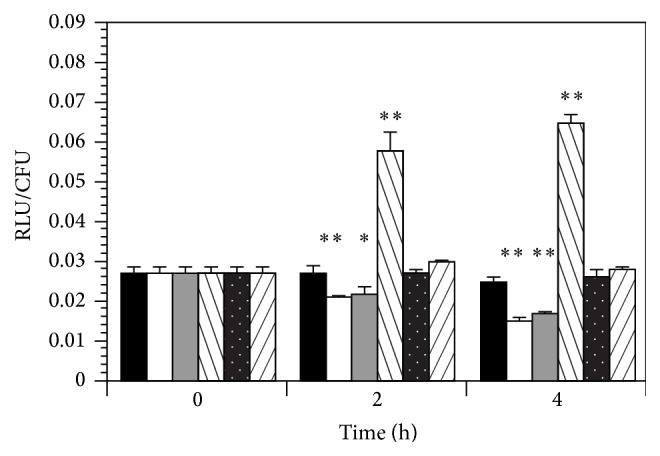
Effects of* fimB* transcription in strain NU149/pWS145-38 containing a* fimB-lux* transcriptional fusion grown in a pH 5.5 or 7 environment mixed with plain Sepharose beads, mannose-coated Sepharose beads, of mannose-coated Sepharose beads with 2% free mannose (wt/vol) added. Columns represent NU149 grown in pH 5.5 LB mixed with mannose-coated Sepharose beads (black column), plain Sepharose beads (white column), or mannose-coated Sepharose beads plus 2% free D-mannose (gray column) as well as NU149 grown in pH 7 LB mixed with mannose-coated Sepharose beads (left striped column), plain Sepharose beads (white dots column), or mannose-coated Sepharose beads plus 2% free D-mannose (right striped column). The RLU/CFU were calculated by using a luminometer to measure luminescence, subtracting out the background, and then dividing by viable counts. The data represents that the means ± standard deviations are indicated from at least three separate runs. *∗* equals *P* < 0.05 and *∗∗* equals *P* < 0.0001.

As a follow-up to the LD-RT-PCR results,* fimB* expression was also monitored using strain NU149/pWS145-38 cells grown in pH 5.5 and pH 7.0 LB were mixed with plain Sepharose beads, mannose-coated Sepharose beads, and mannose-coated Sepharose beads with 2% free mannose added. The results indicated that* fimB* expression rose more than twofold after 2 h postmixing with mannose-coated beads at pH 7.0 (RLU/CFU = 0.058) compared to the 0 h time point (RLU/CFU = 0.027; *P* < 0.0001; Figure 2), rising again after 4 h postmix (RLU/CFU = 0.065; *P* < 0.0001). On the other hand, expression remained consistent in a pH 7.0 environment with plain Sepharose beads at 0 h (RLU/CFU = 0.027), 2 h (RLU/CFU = 0.027), and 4 h (RLU/CFU = 0.026; *P* < 0.113 for 0 h versus 4 h). The addition of free mannose blocked the upregulation of* fimB* transcription when the 0 h time point (RLU/CFU = 0.027) was compared to the 4 h time point (RLU/CFU = 0.028, *P* < 0.65). Transcription of* fimB* fell from RLU/CFU = 0.027 at 0 h to RLU/CFU = 0.015 after 4 h when the bacteria were in an acidic environment mixed with plain Sepharose beads (*P* < 0.0001 for 0 h versus 4 h). However, in the tests with mannose-coated beads at pH 5.5,* fimB* expression remained fairly constant across the 0 h, 2 h, and 4 h time points (RLU/CFU = 0.027, 0.027, and 0.025, resp.; *P*< 0.188 for the 0 h versus 4 h). Again, the addition of free mannose to the UPEC-mannose-coated bead mixture resulted in RLU/CFU numbers similar to using plain Sepharose beads (RLU/CFU = 0.027 at 0 h and 0.017 after 4 h). More striking was the comparisons between pH conditions after 4 h mixing. Transcription of* fimB* expression varied markedly between UPEC cells in pH 7 medium mixed with mannose-coated beads compared to cells in pH 5.5 medium mixed with plain Sepharose beads (*P* < 0.0001). These results suggest that binding to mannose receptors helps ameliorate the effects of pH on* fimB* expression in the* E. coli* cells.

### 3.2. Positioning of the Invertible Element Changes after Type 1 Pili Binding to Mannose Receptors

**Figure 3:**
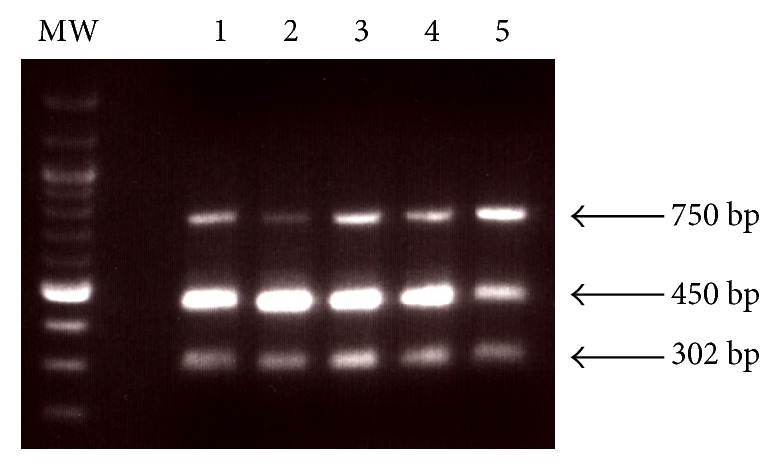
Determination of the* fimS* invertible element orientation in strain NU149 mixed with plain Sepharose beads or mannose-coated Sepharose beads in pH 5.5 or pH 7.0 media by PCR analysis. The PCR analysis was performed with chromosomal DNA isolated from the NU149 cells using the INV and FIMA primers to amplify Phase-ON-oriented DNA (450 bp product), FIME and INV primers to amplify Phase-OFF-oriented DNA (750 bp product), and EcFtsZ1 and EcFtsZ2 primers to amplify the* ftsZ* gene (302 bp product). The products were standardized against the* ftsZ* product using ImageQuant software and the corrected values for both orientations were standardized to the respective 0 h time point. The lanes were loaded as follows: MW = molecular weight standard; lane 1, NU149 at time 0 h; lane 2, NU149 time 24 h, mannose-coated at pH 7.0; lane 3, NU149 time 24 h, plain at 24 h; lane 4, NU149 time 24 h, mannose-coated at pH 5.5; lane 5, NU149 time 24 h, plain at pH 5.5. All PCR products were electrophoresed on 1.5% agarose gels.

Binding of type 1 pili to mannose receptors appeared to shift transcription to favor* fimB* over* fimE*. Since both of the FimB and FimE site-specific recombinases are involved in positioning the* fimA* promoter on the 314 bp* fimS* invertible element to either allow or prevent* fimA* transcription, we predicted that the position of the invertible element would also be affected. To determine whether the position of the invertible element changed after ligand-receptor binding, multiplex PCR amplification with oligonucleotide primers specific for the Phase-ON and Phase-OFF orientations of the* fimS* invertible element [31] as well as the* ftsZ* gene was performed by using chromosomal DNAs extracted from NU149 cells mixed with plain Sepharose beads or mannose-coated beads grown in pH 5.5 and pH 7.0 LB. At the 0 h time point the UPEC population was 8% Phase-OFF. The orientation of the* fimS* invertible element containing the* fimA* promoter caused a twofold decrease in the Phase-OFF orientation (4%) when the UPEC cells were mixed with mannose-coated beads grown in a pH 7.0 environment (Figure 3). A slight shift to the Phase-OFF position was observed when the cells were mixed with plain Sepharose beads in a pH 7.0 environment (11% Phase-OFF). However, there was a significant almost fourfold increase in Phase-OFF positioning (31%) of the UPEC population added to plain Sepharose beads in a pH 5.5 background. A LD-PCR analysis of NU149 cells mixed with plain Sepharose or mannose-coated Sepharose at pH 5.5 and pH 7.0 mirrored the findings shown above (data not shown). These results suggest that attachment of the ligand to its receptor negates the impact that low pH would otherwise have on the orientation of the* fimA* promoter region. Contact between the ligand and receptor appears to favor positioning of the* fimS* invertible element to allow* fimA* transcription, even in an acidic environment.

**Figure 4:**
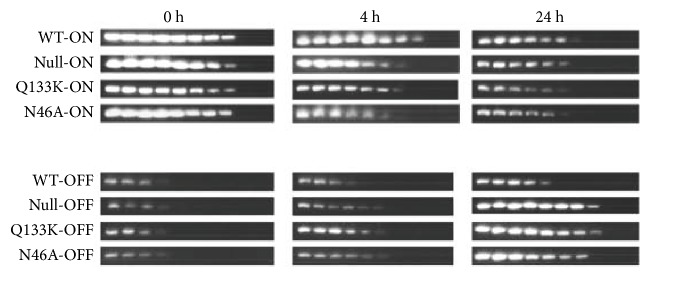
Quantitative determination of the* fimS* invertible element orientation of* E. coli* cells with a plasmid that has the FimH protein represented as wild type (WT), Null, Q133K, or N46A mixed with mannose-coated Sepharose beads (+) for 0 h, 4 h, or 24 h. The PCR analysis was performed with twofold dilutions of chromosomal DNA isolated from the* E. coli* cells using the INV and FIMA primers to amplify Phase-ON-oriented DNA (450 bp product) or FIME and INV primers to amplify Phase-OFF-oriented DNA (750 bp product). All PCR products were electrophoresed on 1.5% agarose gels. The following dilutions of DNA were used: undiluted (lane 1), 1/2 (lane 2), 1/4 (lane 3), 1/8 (lane 4), 1/16 (lane 5), 1/32 (lane 6), 1/64 (lane 7), 1/128 (lane 8), 1/256 (lane 9), and 1/512 (lane 10). The data represent at least three separate runs.

To substantiate that FimH was the ligand involved, several FimH plasmid constructs that have been previously described were used, including a wild type, a null mutant missing the* fimH* gene, a Q133K mutant, and an N46A mutant. Both of the amino acid substitution mutants affected the binding domains of FimH to the mannose receptors [29].* E. coli* cells expressing these plasmids were mixed with mannose-coated Sepharose and the orientation of the invertible element followed after 0 h, 4 h, and 24 h postmixing. After 4 h, the Phase-ON population had dropped 2-fold in the null mutant and Q133K mutant compared to wild type, whereas the N46A mutant had dropped 4-fold (Figure 4). By 24 h, there was a twofold drop in the wild-type strain's Phase-ON population and a fourfold drop when using the FimH mutants. Orientation of the* fimS* element also changed over the time course to be more Phase-OFF. The wild-type strain did not change after 4 h, but all the mutants displayed a fourfold increase in Phase-OFF oriented* fimS* DNA. After 24 h, the wild-type population showed a twofold increase in Phase-OFF oriented DNA, whereas 8- (N46A) to 16-fold (Q133K) increase in Phase-OFF DNA was observed using the FimH mutants. This indicated that FimH binding affected positioning of the* fimS* invertible element.

### 3.3. Type 1 Pilus Expression Changes after Ligand-Receptor Binding

**Figure 5:**
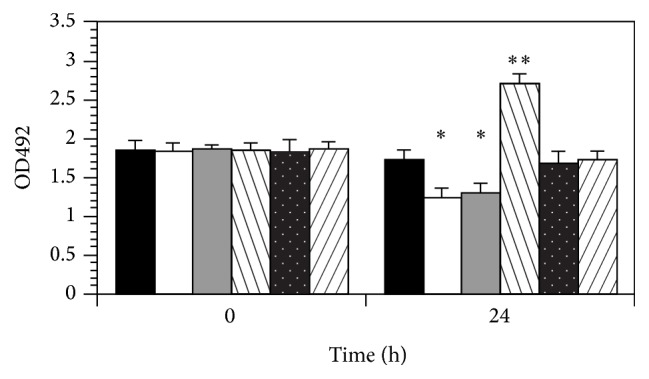
EIA analyses of strain NU149 grown in a pH 5.5 or 7 environment mixed with plain Sepharose beads, mannose-coated Sepharose beads, or mannose-coated Sepharose beads with 2% free mannose (wt/vol) added. Columns represent NU149 grown in pH 5.5 LB mixed with mannose-coated Sepharose beads (black column), plain Sepharose beads (white column), or mannose-coated Sepharose beads plus 2% free D-mannose (gray column) as well as NU149 grown in pH 7 LB mixed with mannose-coated Sepharose beads (left striped column), plain Sepharose beads (white dots column), or mannose-coated Sepharose beads plus 2% free D-mannose (right striped column). Optical densities at 492 (O.D._492_) were determined. The data represents the means ± standard deviations from at least three separate experiments. *∗* equals *P* < 0.05 and *∗∗* equals *P* < 0.0001.

Changes in the levels of* fimB* and* fimE* transcripts combined with alterations in the invertible element suggested that the type 1 pilus expression was conceivably altered after type 1 pilus binding to mannose. To demonstrate variations carried through to the level of type 1 pilus expression, EIAs were done. Strain NU149 cells mixed with mannose-coated Sepharose beads at pH 7.0 showed an increase in type 1 pilus expression after 24 h (2.69) compared with the 0 h time point (1.85; *P* < 0.0001; Figure 5). No significant changes were observed for the cells mixed with plain Sepharose at pH 7.0 (1.83 versus 1.67; *P* < 0.067) or the* E. coli* cells mixed with mannose-coated Sepharose at pH 5.5 (1.85 versus 1.71; *P* < 0.092). However, the* E. coli* cells mixed with plain Sepharose at pH 5.5 after 24 h displayed a significant reduction in type 1 pilus expression (1.23) compared with the 0 h time point (1.82; *P* < 0.0001). When free mannose was added to the mannose-coated beads, type 1 pilus expression dropped to levels close to the EIAs done with plain Sepharose beads, suggesting that the mannose needs to tethered to something (e.g., beads or bladder cell) for the transcriptional activation effect to occur. When free mannose was added to the UPEC cells mixed with mannose-coated beads, type 1 pilus expression dropped to levels close to the EIAs done with UPEC mixed with plain Sepharose beads, suggesting that the mannose needs to be tethered to something for the changes that promote type 1 pilus expression to occur. Thus, not only is transcription of key* fim* genes affected, but the expression of type 1 pili is also affected by binding of FimH to mannose receptors.

## 4. Discussion

Adherence to and invasion into human bladder epithelial cells by UPEC cells is mediated via the type 1 fimbrial adhesin FimH binding to mannose containing residues, such as monosaccharide D-mannose or mannotriose residues found on human bladder epithelial cells [3, 4]. Once FimH attachment to a mannose receptor has been initiated, it has been assumed that physiological changes then occur in the* E. coli* cell. Unfortunately, little has been done to characterize those alterations following a bacterial ligand-receptor interaction. Previous work by Zhang and Normark [33] showed transcriptional activation of a sensor-regulator gene essential for the bacterial iron-starvation response after P fimbriae binding to its receptor, and our recent study demonstrated that capsule assembly gene expression is negatively affected after type 1 piliated UPEC cell binding to mannose receptors [27]. Thus, several changes occur at the transcriptional level within UPEC cells as a consequence of a ligand-receptor binding. However, no one had previously examined the effect of pilus gene expression following attachment of that variety of pilus to its receptor.

In this study, changes in* fim* gene expression were noted following binding of type 1 piliated UPEC bacterial cells to mannose-coated beads. The changes in* fimB* and* fimE* expression led to a shift in the orientation of the* fimS* invertible element containing the* fimA* promoter to favor a Phase-ON positioning, which in turn led to greater expression of type 1 pili on the surface of the UPEC cells. Several mutants were examined (including a* cpxR* mutant strain) to try to elucidate which gene product may be regulating the* fim* genes following binding to mannose receptors, but no gene was linked directly with the regulatory changes affecting* fimB* or* fimE* (data not shown). Thus, a feedback loop appears to be triggered favoring the expression of type 1 pili to maintain the tight adherence generated by the ligand-receptor binding, even in an acidic environment. One possible regulator that could be tied to the FimH-mannose binding changes that we did not examine was OxyR. OxyR is a LysR-type regulator and the* oxyR* gene has been shown to have slightly elevated transcription after FimH mediated adherence to mannose [34]. Expression of type 1 pili in* K. pneumoniae* [35] as well as* Serratia marcescens* [36] was lower in* oxyR* mutant strains compared to the wild-type strains. It is possible that activation of* fimB* is linked to transcriptional activation of the* oxyR* gene following FimH-mannose binding. Attachment of* E. coli* to abiotic surfaces causes physiological changes that favor biofilm formation and subsequently better adherence to the surface [37], and it is very likely that the changes in UPEC cells that occur after the ligand-receptor binding allow the bacteria to sustain the tight adherence.

Certainly, the external environment also plays a role in the expression of the type 1 pili. Lower type 1 pilus expression was observed in UPEC cells found in a pH 5.5 environment compared to a pH 7.0 environment. The human urinary tract is bathed in urine with a pH range between 5.0 and 8.0 [38]. An acidic pH of 5.5 to 6.5 is quite common, which has been shown to lower type 1 pilus expression [24] as well as the expression of other adherence genes [39]. Human urine can also affect type 1 pilus expression [24, 40, 41]. Regulation of the* fim* genes may be affecting type 1 pilus phase variation in the human or murine urinary tract. In the murine kidney, UPEC cells lose their type 1 pili, whereas heavily piliated cells persist in UPEC cells adhering to bladder epithelial cells [26, 28, 42]. These differences in type 1 pilus expression in bacteria found within each organ may be partially attributed to fewer mannose receptors in the kidney compared to the bladder [43–45], coupled with a lower pH and higher osmolarity in the kidney [38]. The loss of type 1 pili in the kidneys may be advantageous for the bacteria because of the greater contact with the immune system in the kidneys, whereas maintaining type 1 pilus expression following the initial attachment to the mannose receptors would be of benefit in the bladder to prevent the bacteria from being washed away by the flow of urine.

Several studies have looked into FimH structure and the subsequent adherence to mannose receptors. A quantitative difference in FimH adherence to mannose residues is the result of structural differences in the* fimH* gene that have arisen naturally [46–49] or through site-specific mutational changes that affect the FimH binding pocket [29]. In this study, we have shown that FimH mutants associated with the mannose-binding pocket [29] affected the FimH ligand binding to mannose residues and subsequent changes in* fimB* and* fimE* transcription within the UPEC cells. Both the Q133K and N46A FimH mutants showed a greater switch to the Phase-OFF orientation after 24 h that mirrored the* fimH* null construct as compared to the wild-type FimH protein. An unperturbed mannose-binding pocket is necessary for FimH to properly bind mannose residues and then turn on a regulatory cascade that leads to changes in* fim* gene expression.

Adherence of* E. coli* to a host cell through a ligand-receptor binding may facilitate cross-talk between the bacterial cell and the host cell that in turn leads to temporal regulation of some of the* fim* genes involved in type 1 pilus expression. Cross-talk between different adherence gene operons affects pilus expression [50, 51] as well as capsule gene expression [27]. Regulation of the* fim* genes appears to be a part of the regulatory cascade that occurs after FimH-mannose binding that may benefit the bacteria differently in each part of the human urinary tract. This cross-talk may allow the UPEC cells to adapt readily to changing environments within the human body to allow bacterial cell survival in a range of harsh environments, including the human urinary tract.

## 5. Conclusion

The binding of FimH to its tethered mannose receptor causes transcriptional activation of the* fimB* gene that leads to increased type 1 pili expression.

## Data Availability

All data that support the findings of this study are available from the corresponding author upon reasonable request.
